# Characterization of *bla*_CTX-M_ sequences of Indian origin and thirteen uropathogenic *Escherichia coli* isolates resistant to multiple antibiotics

**DOI:** 10.1186/s13104-018-3735-5

**Published:** 2018-08-31

**Authors:** Shivakumara Siddaramappa, Karthik Pullela, Bhagya Thimmappa, Ranjan Devkota, Rani Bajaj, Bhavani Manivannan, Niranjana Mahalingam, Bulagonda Eswarappa Pradeep

**Affiliations:** 1 Institute of Bioinformatics and Applied Biotechnology, Biotech Park, Electronic City, Bengaluru, Karnataka India; 2 Sri Sathya Sai Institute of Higher Learning, Vidyagiri, Prasanthi Nilayam, Andhra Pradesh India; 3 Sri Sathya Sai Institute of Higher Medical Sciences, Prasanthigram, Andhra Pradesh India

**Keywords:** *Enterobacteriaceae*, *Escherichia coli*, Resistance, ESBL, *bla*_CTX-M-15_, HGT, India

## Abstract

**Objectives:**

ESBL-producing isolates of the *Enterobacteriaceae* occur throughout the world. The objectives of this study were to characterize uropathogenic *Escherichia coli* isolated at a tertiary care hospital in southern India, and shed light on *bla*_CTX-M_ sequences of Indian origin.

**Results:**

A cohort of 13 urinary isolates of *E. coli* (obtained from patients at the Sri Sathya Sai Institute of Higher Medical Sciences, Prasanthigram, Andhra Pradesh, India) were characterized and found to be resistant to multiple antibiotics, including extended-spectrum cephalosporins. All 13 isolates contained *bla*_CTX-M-15_, and many of them transferred this genotype to at least one laboratory strain of *E. coli* after conjugation. Analyses of *bla*_CTX-M-15_ sequences (n = 141) of Indian origin showed that > 85% of them were obtained from bacteria not associated with the urinary tract, and that *E. coli* isolates account for majority of all *bla*_CTX-M-15_-carrying bacteria reported from India. Other types of *bla*_CTX-M_ appear to be rare in India, since only six such sequences were reported as of July 2015. The results indicate that ‘selection pressure’ exerted by extended-spectrum cephalosporins may have stabilized the *bla*_CTX-M-15_ genotype among *E. coli* in India. The rarity of other *bla*_CTX-M_ suggests that they lack the survival advantage that *bla*_CTX-M-15_ may have.

**Electronic supplementary material:**

The online version of this article (10.1186/s13104-018-3735-5) contains supplementary material, which is available to authorized users.

## Introduction

Diseases caused by extraintestinal pathogenic *Escherichia coli* (ExPEC) strains among humans are as common and debilitating as those caused by intestinal pathogenic *E. coli* (InPEC) strains [1, 2]. Uropathogenic *E. coli* (UPEC) strains are the most common type of ExPEC that cause urinary tract infections (UTIs) and are a global burden [3, 4]. Most of the UPEC isolates are reported to produce extended spectrum β-lactamases (ESBLs) that limit the choice of therapy [5]. These ESBLs hydrolyze extended-spectrum cephalosporins and the genes encoding them are frequently plasmid and/or mobile element-borne [6–8]. Worldwide, CTX-M is the predominant ESBL type and most of the enzymes within this family mediate resistance to cefotaxime and ceftriaxone [9, 10]. In 2001, a derivative of CTX-M-3 was reported from India that “conferred a higher level of resistance to ceftazidime” and was designated CTX-M-15 [11]. In the first systematic survey of *E. coli* resistant to third-generation cephalosporins in India, it was shown that 73% of the isolates carry *bla*_CTX-M-15_ and that many of these genes were associated with IS*26* [12]. The *bla*_CTX-M-15_ genotype appears to be common among *E. coli* sequence type 131, which is a multidrug-resistant clonal group associated with urinary tract and bloodstream infections [13–15].

Previous studies focused on understanding the features of 16 UPEC ST131 isolates from India revealed that many of them contain large conjugative plasmids encoding the CTX-M-15 β-lactamase [5]. Although the sequences of *bla*_CTX-M-15_ from these 16 ST131 isolates are not available, their diversity was reported to be low [5]. It has also been reported that UTI-associated *E. coli* isolates from HIV patients in India were more likely to contain *bla*_CTX-M-15_ than those from non-HIV patients, and that their ESBL phenotype correlated with the presence of *bla*_CTX-M-15_ [16]. Although several other reports contain details of ESBL-positive *E. coli* isolates from India [17–19], systematic analyses of *bla*_CTX-M-15_ are lacking. The objectives of this study were to characterize ESBL-producing *E. coli* isolated at the Sri Sathya Sai Institute of Higher Medical Sciences, Prasanthigram, Andhra Pradesh, India, and to compare the *bla*_CTX-M_ sequences among these and other bacteria of Indian origin.

## Main text

### Materials and methods

#### Bacterial isolates, conjugation, and sequence analysis

Bacterial isolates (n = 70) were obtained from the clinical microbiology section of the Sri Sathya Sai Institute of Higher Medical Sciences, Prasanthigram, Andhra Pradesh, India. These isolates represented pure cultures derived from non-HIV patients. The isolates were identified using VITEK 2 systems version 06.01 (bioMérieux). Antibiotic susceptibility testing of the isolates was performed using VITEK-2 AST-N280 cards (bioMérieux) and the results were interpreted based on the clinical and laboratory standards institute guidelines (CLSI M100-S24; 2014). Bacterial chromosomal DNA, obtained after heat lysis of pure cultures [20], served as the template for amplification of gene segments. Isolates were screened for *bla*_CTX-M_, *bla*_SHV_, and *bla*_TEM_ genes using PCR as described previously [21–24]. Primer sequences and thermocycling protocol are provided in Additional file 1. Products amplified by PCR were gel purified, cloned, and sequenced.

Horizontal transferability of genes/elements encoding antibiotic resistance was tested using three different *E. coli* recipient strains in conjugation experiments. These include strain B (a component of the HiPer^®^ bacterial conjugation teaching kit supplied by Hi Media Laboratories Pvt Ltd., Mumbai, India), strain XL1-Blue (supplied by the erstwhile Stratagene, La Jolla, CA), and strain BL-21 CodonPlus (also supplied by Stratagene), which are resistant to streptomycin, tetracycline, and chloramphenicol, respectively. Conjugation protocol is provided in Additional file 1.

Clustal omega (http://www.ebi.ac.uk/Tools/msa/clustalo/) was used to obtain an initial alignment of the *bla*_CTX-M_ sequences. This alignment was used as a guide to manually trim the sequences when they were of different lengths. Progressive alignment of DNA sequences was performed using ClustalW with default parameters. Phylogeny was reconstructed using the maximum likelihood method (with 1000 bootstrap replicates) and the Tamura-Nei substitution/scoring matrix in MEGA 6.0.

### Results

#### Antibiotic resistance

Thirteen apparently disparate isolates were selected for further study based on them containing plasmids of various sizes (data not shown). The isolates were confirmed as *E. coli* using the amplification and sequencing of their 16S rDNA genes. The sources of these 13 isolates and the clinical complaints/diagnosis of the patients are listed in Additional file 2. Antibiotic sensitivity testing in the clinical laboratory using VITEK-2 showed that all 13 isolates were resistant to extended spectrum β-lactam antibiotics such as cefazolin, ceftriaxone, and/or cefepime, indicating that they were capable of producing ESBLs (Table 1). However, most of these isolates were sensitive to ertapenem, imipenem, and/or meropenem, indicating that they were carabapenemase negative (Table 1). All 13 isolates consistently tested positive for *bla*_CTX-M_, but negative for *bla*_SHV_ and *bla*_TEM_, in PCR experiments.

**Table 1:** Antibiotic sensitivity testing of urinary isolates of *E. coli* in the clinical laboratory using VITEK-2 AST-N280 cards

Designation of the isolate	Cefazolin		Ceftriaxone		Cefepime		Ertapenem		Imipenem		Meropenem		Amikacin		Ciprofloxacin		Nitrofurantoin	
	MIC	INT	MIC	INT	MIC	INT	MIC	INT	MIC	INT	MIC	INT	MIC	INT	MIC	INT	MIC	INT
P8	≥ 64	R	≥ 64	R	16	R	≤ 0.5	S	≤ 1	S	≤ 0.25	S	≤ 2	S	≥ 4	R	64	I
P12	≥ 64	R	≥ 64	R	2	R	≤ 0.5	S	≤ 1	S	≤ 0.25	S	≤ 2	S	≥ 4	R	128	R
P19	≥ 64	R	≥ 64	R	32	R	≤ 0.5	S	≤ 1	S	≤ 0.25	S	8	S	≥ 4	R	≤ 16	S
P20	≥ 64	R	≥ 64	R	8	R	≤ 0.5	S	≤ 1	S	≤ 0.25	S	16	S	≥ 4	R	64	I
P28A	≥ 64	R	≥ 64	R	8	R	≤ 0.5	S	≤ 1	S	≤ 0.25	S	16	S	≥ 4	R	128	R
P45	≥ 64	R	≥ 64	R	32	R	≤ 0.5	S	≤ 1	S	≤ 0.25	S	8	S	≥ 4	R	64	I
Q41A	≥ 64	R	≥ 64	R	32	R	≤ 0.5	S	≤ 1	S	≤ 0.25	S	16	S	≥ 4	R	64	I
Q42B	≥ 64	R	≥ 64	R	8	R	≤ 0.5	S	≤ 1	S	≤ 0.25	S	16	S	≥ 4	R	≤ 16	S
Q57	≥ 64	R	16	R	4	R	4	I	≤ 1	S	≤ 0.25	S	≤ 2	S	≤ 0.25	S	≥ 512	R
Q66	≥ 64	R	≥ 64	R	8	R	≥ 64	R	≤ 1	S	≤ 0.25	S	8	S	≥ 4	R	≤ 16	S
Q72	≥ 64	R	≥ 64	R	≥ 64	R	≤ 0.5	S	≤ 1	S	≤ 0.25	S	4	S	2	I	32	S
Q76A	NT	NT	≥ 64	R	32	R	≤ 0.5	S	≤ 1	S	≤ 0.25	S	16	S	≥ 4	R	64	I
Q76B	NT	NT	≥ 64	R	16	R	≤ 0.5	S	≤ 1	S	≤ 0.25	S	16	S	≥ 4	R	64	I

*MIC* minimum inhibitory concentration (µg/ml), *INT* interpretation, *R* resistant, *S* sensitive, *I* intermediate, *NT* not tested

The 13 isolates shown in Table 1 were further screened in the research laboratory for their sensitivity to six antibiotics using LB agar plates. Laboratory strains of *E. coli* (B, DH5α, XL1-Blue, and BL-21 CodonPlus) were used as controls in these tests. All 13 isolates were resistant to ampicillin and nalidixic acid (100 µg/ml each; data not shown). Except isolate Q57, all others were also resistant to kanamycin (50 µg/ml; data not shown). Data is shown in Table 2 for four isolates (Q41A, Q66, Q72, and Q76A) that were sensitive to streptomycin (100 µg/ml) and tetracycline (60 µg/ml), for three isolates (P8, Q42, and Q76B) that were sensitive to streptomycin but resistant to tetracycline, and for one isolate (Q57) that was sensitive to tetracycline but resistant to streptomycin. Except isolates P20, P28A, and P45, all others were sensitive to chloramphenicol (Table 2). These results suggest that the 13 isolates are sufficiently disparate and non-clonal.

**Table 2:** Antibiotic sensitivity testing of urinary isolates of *E. coli* in the research laboratory and the results of conjugation experiments

Designation of the isolate	*bla*_CTX-M-15_ GenBank Accession	Antibiotic sensitivity			Conjugation with *E. coli*		
		Streptomycin (100 µg/ml)	Tetracycline (60 µg/ml)	Chloramphenicol (34 µg/ml)	Strain B (Str^R^ + Amp^R^)	Strain XL1-Blue (Tet^R^ + Amp^R^)	Strain BL-21 CodonPlus (Cm^R^ + Amp^R^)
P8	KT956436	S	R	S	Positive	Cannot be tested	Positive
P12	KY568704	R	R	S	Cannot be tested	Cannot be tested	Positive
P19	KT956438	R	R	S	Cannot be tested	Cannot be tested	Positive
P20	KY568702	R	R	R	Cannot be tested	Cannot be tested	Cannot be tested
P28A	KY568703	R	R	R	Cannot be tested	Cannot be tested	Cannot be tested
P45	KT956439	R	R	R	Cannot be tested	Cannot be tested	Cannot be tested
Q41A	KU987443	S	S	S	Positive	Positive	Positive
Q42B	KX009505	S	R	S	Positive	Cannot be tested	Positive
Q57	KT956440	R	S	S	Cannot be tested	Positive	Positive
Q66	KT956441	S	S	S	Positive	Positive	Positive
Q72	KT956442	S	S	S	Positive	Positive	Positive
Q76A	KX009504	S	S	S	Positive	Positive	Positive
Q76B	KU987444	S	R	S	Positive	Cannot be tested	Positive

#### Horizontal transfer of bla_CTX-M_

Conjugation was performed based on the antibiotic sensitivity of the 10 donor/clinical isolates using appropriate recipient strains. Isolates P8, Q41A, Q42, Q66, Q72, Q76A, or Q76B were conjugated with *E. coli* strain B as the recipient and produced transconjugants that were resistant to ampicillin and streptomycin (Table 2). Furthermore, transconjugants obtained using isolates P8, Q42, or Q76B were resistant to tetracycline, and transconjugants obtained using isolates P8, Q42, Q66, Q72, Q76A, and Q76B were resistant to nalidixic acid. These results indicate that in some clinical isolates, different resistance determinants were co-located and/or co-transferable. Such mobilizable elements encoding resistance to extended spectrum β-lactam antibiotics and several other antibacterial drugs have been reported among ST131 isolates from India [5].

Isolates Q41A, Q57, Q66, Q72, and Q76A were conjugated with *E. coli* strain XL1-Blue as the recipient and produced transconjugants that were resistant to ampicillin and tetracycline (Table 2). Interestingly, strain XL1-Blue transconjugants obtained using isolates Q57, Q72, or Q76A readily transferred ampicillin resistance to strain B after secondary conjugation (data not shown). However, none of the strain B transconjugants that were sensitive to tetracycline could transfer ampicillin resistance to strain XL1-Blue after secondary conjugation. This indicates that some strain XL1-Blue transconjugants, unlike those of strain B, possess the traits to serve as donors, and that conjugative elements borne by some strains (such as Q41A and Q66) may require host-derived factors for their efficient mobilization.

*Escherichia coli* strain BL-21 CodonPlus is generally used for protein expression and contains a ColE1-compatible pACYC-based plasmid (~ 3.5 kb) that confers chloramphenicol resistance. The suitability of this strain as a recipient in conjugation experiments has not been reported. Isolates P8, P12, P19, Q41A, Q42, Q57, Q66, Q72, Q76A, or Q76B were conjugated with strain BL-21 CodonPlus as the recipient and produced transconjugants that were resistant to ampicillin and chloramphenicol (Table 2). The presence of *bla*_CTX-M_ in ampicillin-resistant strain B, XL-1 Blue, or BL-21 CodonPlus transconjugants was confirmed using PCR (data not shown).

#### Epidemiology of bla_CTX-M_ in India

**Fig. 1:**
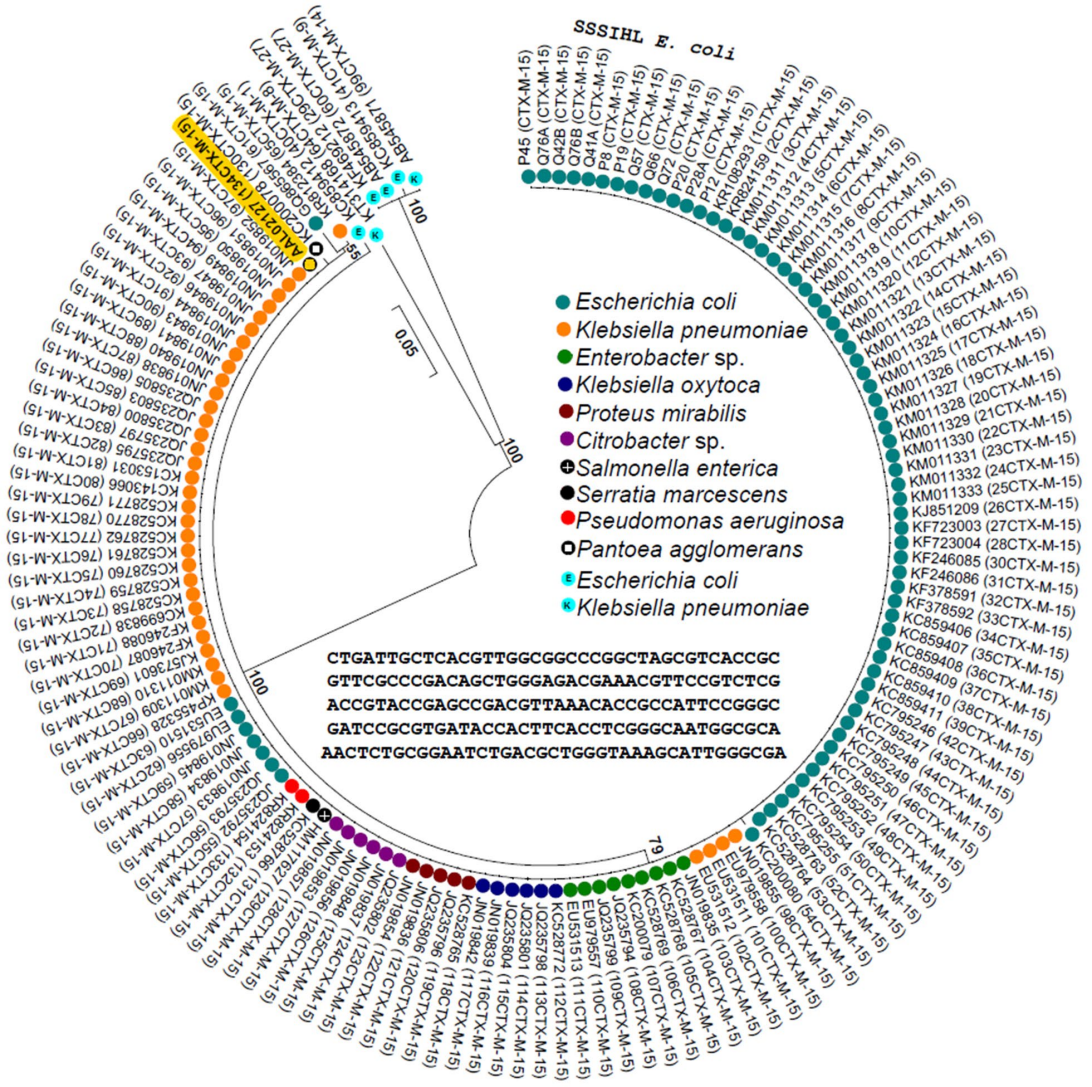
Phylogenetic tree based on 147 *bla*_CTX-M_ sequences (191 bp). The unrooted tree was constructed using the maximum likelihood method in MEGA 6.0. Bootstrap values of 1000 replicates are indicated as numbers out of 100 at the nodes. Scale bar shows the number of nucleotide substitutions per site. ‘SSSIHL *E. coli*’ refer to the *bla*_CTX-M-15_ sequences from the 13 isolates characterized in this study whose accession numbers are given in Table 2. The accession numbers of 134 *bla*_CTX-M_ sequences (128 of *bla*_CTX-M-15_, two of *bla*_CTX-M-27_, one each of *bla*_CTX-M-1_, *bla*_CTX-M-8_, *bla*_CTX-M-9_, and *bla*_CTX-M-14_) are shown in the figure (and in Additional file 3). Colors used for representing various bacterial genera/species are explained within the figure. The first *bla*_CTX-M-15_ sequence with the accession number AAL02127 submitted in July 2001 is highlighted in orange. A representative 191 bp *bla*_CTX-M-15_ sequence (from isolate Q76A) is also shown within the figure

From each of the 13 isolates, ~ 541 bp fragment of the *bla*_CTX-M_ gene was amplified by PCR, cloned, and sequenced. Sequence comparison by blastn indicated that all 13 isolates carried a *bla*_CTX-M-15_ gene (GenBank accession numbers are shown in Table 2). For further comparison, a total of 133 *bla*_CTX-M_ sequences deposited in GenBank from various parts of India from February 2008 to July 2015 (plus the first *bla*_CTX-M-15_ sequence with the accession number AAL02127 submitted in July 2001) were obtained (Additional file 3). Since the sequences varied in length, they were trimmed to obtain a 191 bp internal fragment after multiple sequence alignment. A maximum likelihood phylogenetic tree (with 1000 bootstrap replicates) constructed using 147 *bla*_CTX-M_ sequences showed that 96% of them (141/147, including 13 from the isolates characterized in this study) cluster together (Fig. 1). Among the 141 sequences that were compared in Fig. 1, the 191 bp internal fragment was almost identical, indicating the genetic homogeneity of *bla*_CTX-M-15_. Not surprisingly, 51% (72/141) of the sequences were from *E. coli* isolates, and 26% (37/141) were from *Klebsiella pneumoniae* isolates. Interestingly, two sequences were from *Pseudomonas aeruginosa* isolates causing ocular infections (Additional file 3). Furthermore, only 13% of the sequences (19/141) were from *E. coli* cultured using urine samples. Other types of *bla*_CTX-M_ appear to be rare in India, since only one sequence for each of *bla*_CTX-M-1_, *bla*_CTX-M-8_, *bla*_CTX-M-9_, and *bla*_CTX-M-14_ and two sequences of *bla*_CTX-M-27_ were found in the GenBank database. These *bla*_CTX-M_ sequences were also from *E. coli* and *K. pneumoniae* (Fig. 1 and Additional file 3).

### Discussion

In a previous study assessing the drug resistance of 16 ESBL-producing *E. coli* isolates, it was reported that 84% were resistant to ciprofloxacin [5]. Furthermore, resistance to extended spectrum β-lactam antibiotics and fluoroquinolones among UTI-associated *E. coli* isolates from HIV patients was reported to be significantly higher than those from non-HIV patients [16]. Co-resistance to ciprofloxacin and at least one extended spectrum β-lactam antibiotic has also been reported among *E. coli* isolates from pregnant women [17]. In the current study, eleven isolates (85%) were found to be resistant to ciprofloxacin. These results suggest that fluoroquinolones are not a good choice in the Indian scenario for treating ESBL-producing *E. coli*. Based on previous reports [5] and current work, it appears that at least 70% of UTI-associated ESBL-producing *E. coli* isolates could be sensitive to chloramphenicol. This sensitivity may be due to infrequent use of chloramphenicol in humans, and provides a choice to clinicians to prescribe the drug for life-threatening infections caused by *E. coli*. The results of the conjugation experiments indicate that the *bla*_CTX-M-15_ gene was borne on a mobile genetic element in each donor strain. It was obvious from this study that members of the *Enterobacteriaceae* are the majority among all potentially ESBL-producing bacteria of clinical relevance in India. It appears that the genetic and epidemiological features of CTX-M β-lactamases have not changed much since the first systematic survey reported in 2006 [12]. Sequence analyses indicated that *E. coli* isolates account for at least 50% of all *bla*_CTX-M-15_ carrying bacteria, and that other types of *bla*_CTX-M_ are rare in India. Therefore, there is likely a “strong selection pressure” for the maintenance and dispersal of the *bla*_CTX-M-15_ genotype among pathogenic *E. coli* in India. In view of this, diagnostic laboratories should routinely test clinical isolates of *E. coli* for *bla*_CTX-M-15_, and hospitals should develop and implement an antibiotic stewardship program to reverse the trend.

## Limitations

This work relied on few ESBL-positive UPEC isolates from a single hospital. Studies using many isolates from different hospitals could provide broader insights into ESBL-positive UPEC in India. For conjugation experiments, three different recipient strains of *E. coli* were used. A single recipient *E. coli* strain could help in better assessment of horizontal gene transfer. This study used *bla*_CTX-M-15_ sequences available in GenBank regardless of the origins of the host strains. Future studies could look into the distribution of *bla*_CTX-M-15_ among clinical and non-clinical strains.

## Additional files

**Additional file 1.** Primer sequences and thermocycling protocol.

**Additional file 2.** Clinical isolates used in this study and their sources.

**Additional file 3.** Details of *bla*_CTX-M_ sequences deposited in GenBank from various parts of India that were used in this study.

## Abbreviations

*bla*: β-lactamase
CTX: cefotaxime
ESBL: extended spectrum β-lactamase
HGT: horizontal gene transfer
