# Supplementary material for: Characterization of blaCTX-M sequences of Indian origin and thirteen uropathogenic Escherichia coli isolates resistant to multiple antibiotics
Source: BMC Res Notes. 2018 Aug 31;11:630. doi: 10.1186/s13104-018-3735-5 (PMC6119312; doi:10.1186/s13104-018-3735-5)
Supplement: Supplementary file 1 — Additional file 1. Primer sequences and thermocycling protocol. [file 13104_2018_3735_MOESM1_ESM.pdf]

Primers for PCR used in the current study and their characteristics			
Target gene	Forward primer (Tm°C)	Reverse primer (Tm°C)	Expected amplicon size
16S rDNA	5'-AGAGTTTGATCMTGGCTCAG-3'(53.2)	5'-TACGGYTACCTTGTTACGACTT-3'(54.6)	~1500 bp
bla$_{CTX-M}$	5'-SCSATGTGCAGYACCAGTAA-3'(56.4)	5'-CCGARATATGRTTGGTGGTG-3'(56.4)	~550bp
bla$_{SHV}$	5'-TTATCTCCCTGTTAGCCACC-3'(53.8)	5'-GATTTGCTGATTTCGCTCGG-3'(54.6)	~800 bp
bla$_{TEM}$	5'-TCGGGGAAATGTGCGCG-3'(59.1)	5'-TGCTTAATCAGTGAGGCACC-3'(55.0)	~1000 bp

Thermocycling conditions for PCR amplification of DNA using primers listed above			
STEP	TEMPERATURE	TIME	NUMBER OF CYCLES
Initial Denaturation	95°C	5 minutes	1
Denaturation	94°C	45 seconds	30
Annealing	54°C	1 minute	
Extension	72°C	1 minute	
Final extension	72°C	5 minutes	1
Hold	4°C	Indefinite	

Conjugation protocol

Each donor and recipient strain was cultured using Luria Bertani (LB) broth to an OD_{600} of 0.8–0.9 in the presence of appropriate antibiotics (100 µg/ml of ampicillin for donors; 100 µg/ml steptomycin, 60 µg/ml tetracycline, or 34 µg/ml chloramphenicol for recipients). The donor and recipient strains were mixed (1:1 ratio,100 µl each) in a test tube and incubated at 37°C for 2 hours without shaking. Cells were recovered by adding 2 ml sterile LB broth and incubated at 37°C for 2 hours with shaking. Transconjugants (defined as host cells that received a mobile element) were selected by plating 100 µl of cells on LB agar plates containing 100 µg/ml of ampicillin and one of the three antibiotics (100 µg/ml steptomycin, 60 µg/ml tetracycline, or 34 µg/ml chloramphenicol) based on the recipient used.
