# Supplementary material for: Characterization of blaCTX-M sequences of Indian origin and thirteen uropathogenic Escherichia coli isolates resistant to multiple antibiotics
Source: BMC Res Notes. 2018 Aug 31;11:630. doi: 10.1186/s13104-018-3735-5 (PMC6119312; doi:10.1186/s13104-018-3735-5)
Supplement: Supplementary file 2 — Additional file 2. Clinical isolates used in this study and their sources. [file 13104_2018_3735_MOESM2_ESM.pdf]

Additional file 2. Clinical isolates used in this study and their sources

Designation of the isolate	Specimen from which the isolate was cultured	Age/sex of the patient	Clinical complaint/diagnosis
P8	Midstream urine	66/F	Cataract
P12	Midstream urine	68/F	Renal pelvic stone
P19	Midstream urine	55/M	Coronary artery disease and hypertension
P20	Midstream urine	69/M	Emphysematous pyelonephritis
P28A	Midstream urine	32/M	Left ureteric calculus
P45	Midstream urine	79/F	Right solitary kidney with pelvic calculus
Q41A	Midstream urine	64/M	Benign prostatic hyperplasia
Q42B	Midstream urine	41/M	Pelvi-ureteric junction obstruction
Q57	Supra pubic catheter urine	26/M	Pelvic fracture urethral distraction defect
Q66	Midstream urine	12/M	Hypopsadias
Q72	Midstream urine	21/M	Cardiomegaly
Q76A	Midstream urine	57/F	Genito-urinary tuberculosis
Q76B	Midstream urine	57/F	Genito-urinary tuberculosis
