# Supplementary material for: Characterization of blaCTX-M sequences of Indian origin and thirteen uropathogenic Escherichia coli isolates resistant to multiple antibiotics
Source: BMC Res Notes. 2018 Aug 31;11:630. doi: 10.1186/s13104-018-3735-5 (PMC6119312; doi:10.1186/s13104-018-3735-5)
Supplement: Supplementary file 3 — Additional file 3. Details of blaCTX-M sequences deposited in GenBank from various parts of India that were used in this study. [file 13104_2018_3735_MOESM3_ESM.pdf]

Additional file 3.
Details of *bla*$_{CTX-M}$ sequences deposited in GenBank from various parts of India that were used in this study

Sl. no.	Accession no.	Deposit date	Host strain	Source of isolation	Genotype	City and State of origin
1	KR108293	15-APR-2015	*Escherichia coli*	Duck fecal sample	*bla*$_{CTX-M-15}$	Kolkata, West Bengal
2	KR824159	16-MAY-2015	*Escherichia coli*	Urine	*bla*$_{CTX-M-15}$	Kolkata, West Bengal
3	KM011311	16-JUN-2014	*Escherichia coli*	Meat	*bla*$_{CTX-M-15}$	Nongpoh, Meghalaya
4	KM011312	16-JUN-2014	*Escherichia coli*	Water	*bla*$_{CTX-M-15}$	Nongpoh, Meghalaya
5	KM011313	16-JUN-2014	*Escherichia coli*	Water	*bla*$_{CTX-M-15}$	Nongpoh, Meghalaya
6	KM011314	16-JUN-2014	*Escherichia coli*	Meat	*bla*$_{CTX-M-15}$	Nongpoh, Meghalaya
7	KM011315	16-JUN-2014	*Escherichia coli*	Meat	*bla*$_{CTX-M-15}$	Nongpoh, Meghalaya
8	KM011316	16-JUN-2014	*Escherichia coli*	Water	*bla*$_{CTX-M-15}$	Nongpoh, Meghalaya
9	KM011317	16-JUN-2014	*Escherichia coli*	Water	*bla*$_{CTX-M-15}$	Nongpoh, Meghalaya
10	KM011318	16-JUN-2014	*Escherichia coli*	Water	*bla*$_{CTX-M-15}$	Nongpoh, Meghalaya
11	KM011319	16-JUN-2014	*Escherichia coli*	Meat	*bla*$_{CTX-M-15}$	Nongpoh, Meghalaya
12	KM011320	16-JUN-2014	*Escherichia coli*	Meat	*bla*$_{CTX-M-15}$	Nongpoh, Meghalaya
13	KM011321	16-JUN-2014	*Escherichia coli*	Meat	*bla*$_{CTX-M-15}$	Nongpoh, Meghalaya
14	KM011322	16-JUN-2014	*Escherichia coli*	Meat	*bla*$_{CTX-M-15}$	Nongpoh, Meghalaya
15	KM011323	16-JUN-2014	*Escherichia coli*	Meat	*bla*$_{CTX-M-15}$	Nongpoh, Meghalaya
16	KM011324	16-JUN-2014	*Escherichia coli*	Meat	*bla*$_{CTX-M-15}$	Nongpoh, Meghalaya
17	KM011325	16-JUN-2014	*Escherichia coli*	Meat	*bla*$_{CTX-M-15}$	Nongpoh, Meghalaya
18	KM011326	16-JUN-2014	*Escherichia coli*	Meat	*bla*$_{CTX-M-15}$	Nongpoh, Meghalaya
19	KM011327	16-JUN-2014	*Escherichia coli*	Meat	*bla*$_{CTX-M-15}$	Nongpoh, Meghalaya
20	KM011328	16-JUN-2014	*Escherichia coli*	Unknown	*bla*$_{CTX-M-15}$	Nongpoh, Meghalaya
21	KM011329	16-JUN-2014	*Escherichia coli*	Unknown	*bla*$_{CTX-M-15}$	Nongpoh, Meghalaya
22	KM011330	16-JUN-2014	*Escherichia coli*	Unknown	*bla*$_{CTX-M-15}$	Nongpoh, Meghalaya
23	KM011331	16-JUN-2014	*Escherichia coli*	Unknown	*bla*$_{CTX-M-15}$	Nongpoh, Meghalaya
24	KM011332	16-JUN-2014	*Escherichia coli*	Unknown	*bla*$_{CTX-M-15}$	Nongpoh, Meghalaya
25	KM011333	16-JUN-2014	*Escherichia coli*	Unknown	*bla*$_{CTX-M-15}$	Nongpoh, Meghalaya
26	KJ851209	20-MAY-2014	*Escherichia coli*	Fecal sample	*bla*$_{CTX-M-15}$	Cuttack, Odhisha
27	KF723003	14-SEP-2013	*Escherichia coli*	Urine	*bla*$_{CTX-M-15}$	Madurai, Tamil Nadu
28	KF723004	17-SEP-2013	*Escherichia coli*	Urine	*bla*$_{CTX-M-15}$	Madurai, Tamil Nadu
29[1]	KF469212	25-JUL-2013	*Escherichia coli*	Urine	*bla*$_{CTX-M-27}$	Kolkata, West Bengal
30	KF246085	14-JUN-2013	*Escherichia coli*	Unknown	*bla*$_{CTX-M-15}$	Chennai, Tamil Nadu

31	KF246086	14-JUN-2013	*Escherichia coli*	Unknown	*bla*$_{CTX\text{-}M\text{-}15}$	Chennai, Tamil Nadu
32	KF378591	10-JUL-2013	*Escherichia coli*	Urine	*bla*$_{CTX\text{-}M\text{-}15}$	Chennai, Tamil Nadu
33	KF378592	10-JUL-2013	*Escherichia coli*	Urine	*bla*$_{CTX\text{-}M\text{-}15}$	Chennai, Tamil Nadu
34	KC859406	05-APR-2013	*Escherichia coli*	Unknown	*bla*$_{CTX\text{-}M\text{-}15}$	Varanasi, Uttar Pradesh
35	KC859407	05-APR-2013	*Escherichia coli*	Unknown	*bla*$_{CTX\text{-}M\text{-}15}$	Varanasi, Uttar Pradesh
36	KC859408	05-APR-2013	*Escherichia coli*	Unknown	*bla*$_{CTX\text{-}M\text{-}15}$	Varanasi, Uttar Pradesh
37	KC859409	05-APR-2013	*Escherichia coli*	Unknown	*bla*$_{CTX\text{-}M\text{-}15}$	Varanasi, Uttar Pradesh
38	KC859410	05-APR-2013	*Escherichia coli*	Unknown	*bla*$_{CTX\text{-}M\text{-}15}$	Varanasi, Uttar Pradesh
39	KC859411	05-APR-2013	*Escherichia coli*	Unknown	*bla*$_{CTX\text{-}M\text{-}15}$	Varanasi, Uttar Pradesh
40[1]	KC859412	05-APR-2013	*Escherichia coli*	Unknown	*bla*$_{CTX\text{-}M\text{-}1}$	Varanasi, Uttar Pradesh
41[1]	KC859413	05-APR-2013	*Escherichia coli*	Unknown	*bla*$_{CTX\text{-}M\text{-}9}$	Varanasi, Uttar Pradesh
42	KC795246	19-MAR-2013	*Escherichia coli*	Perianal swab	*bla*$_{CTX\text{-}M\text{-}15}$	Bengaluru, Karnataka
43	KC795247	19-MAR-2013	*Escherichia coli*	Perianal swab	*bla*$_{CTX\text{-}M\text{-}15}$	Bengaluru, Karnataka
44	KC795248	19-MAR-2013	*Escherichia coli*	Perianal swab	*bla*$_{CTX\text{-}M\text{-}15}$	Bengaluru, Karnataka
45	KC795249	19-MAR-2013	*Escherichia coli*	Perianal swab	*bla*$_{CTX\text{-}M\text{-}15}$	Bengaluru, Karnataka
46	KC795250	19-MAR-2013	*Escherichia coli*	Perianal swab	*bla*$_{CTX\text{-}M\text{-}15}$	Bengaluru, Karnataka
47	KC795251	19-MAR-2013	*Escherichia coli*	Perianal swab	*bla*$_{CTX\text{-}M\text{-}15}$	Bengaluru, Karnataka
48	KC795252	19-MAR-2013	*Escherichia coli*	Perianal swab	*bla*$_{CTX\text{-}M\text{-}15}$	Bengaluru, Karnataka
49	KC795253	19-MAR-2013	*Escherichia coli*	Perianal swab	*bla*$_{CTX\text{-}M\text{-}15}$	Bengaluru, Karnataka
50	KC795254	19-MAR-2013	*Escherichia coli*	Perianal swab	*bla*$_{CTX\text{-}M\text{-}15}$	Bengaluru, Karnataka
51	KC795255	19-MAR-2013	*Escherichia coli*	Perianal swab	*bla*$_{CTX\text{-}M\text{-}15}$	Bengaluru, Karnataka
52	KC528763	18-JAN-2013	*Escherichia coli*	Vitreous humor	*bla*$_{CTX\text{-}M\text{-}15}$	Chennai, Tamil Nadu
53	KC528764	18-JAN-2013	*Escherichia coli*	Intra ocular lens	*bla*$_{CTX\text{-}M\text{-}15}$	Chennai, Tamil Nadu
54	KC200080	18-NOV-2012	*Escherichia coli*	Corneal rim	*bla*$_{CTX\text{-}M\text{-}15}$	Tirunelveli, Tamil Nadu
55	JQ235792	05-DEC-2011	*Escherichia coli*	Vitreous humour	*bla*$_{CTX\text{-}M\text{-}15}$	Chennai, Tamil Nadu
56	JQ235793	05-DEC-2011	*Escherichia coli*	Intra ocular lens	*bla*$_{CTX\text{-}M\text{-}15}$	Chennai, Tamil Nadu
57	JN019833	24-MAY-2011	*Escherichia coli*	Corneal rim	*bla*$_{CTX\text{-}M\text{-}15}$	Chennai, Tamil Nadu
58	JN019834	24-MAY-2011	*Escherichia coli*	Conjunctival swab	*bla*$_{CTX\text{-}M\text{-}15}$	Chennai, Tamil Nadu
59	JN019845	24-MAY-2011	*Escherichia coli*	Eviscerated material	*bla*$_{CTX\text{-}M\text{-}15}$	Chennai, Tamil Nadu
60[1]	AB545872	04-FEB-2010	*Escherichia coli*	Neonatal stool	*bla*$_{CTX\text{-}M\text{-}27}$	Kolkata, West Bengal
61	GQ865567	02-SEP-2009	*Escherichia coli*	Unknown	*bla*$_{CTX\text{-}M\text{-}15}$	Aligarh, Uttar Pradesh
62	EU979556	01-JUL-2008	*Escherichia coli*	Endotracheal secretion	*bla*$_{CTX\text{-}M\text{-}15}$	Chennai, Tamil Nadu

63	EU531510	23-FEB-2008	*Escherichia coli*	Urine	*bla*$_{\text{CTX-M-15}}$	Chennai, Tamil Nadu
64[1]	KT314168	17-JUL-2015	*Klebsiella pneumoniae*	Bovine milk	*bla*$_{\text{CTX-M-8}}$	Kolkata, West Bengal
65	KR812384	07-MAY-2015	*Klebsiella pneumoniae*	Milk	*bla*$_{\text{CTX-M-15}}$	Kolkata, West Bengal
66	KP455328	10-JAN-2015	*Klebsiella pneumoniae*	Urine	*bla*$_{\text{CTX-M-15}}$	Kalaburgi, Karnataka
67	KM011309	16-JUN-2014	*Klebsiella pneumoniae*	Meat	*bla*$_{\text{CTX-M-15}}$	Nongpoh, Meghalaya
68	KM011310	16-JUN-2014	*Klebsiella pneumoniae*	Water	*bla*$_{\text{CTX-M-15}}$	Nongpoh, Meghalaya
69	KJ573601	13-MAR-2014	*Klebsiella pneumoniae*	Urine	*bla*$_{\text{CTX-M-15}}$	Madurai, Tamil Nadu
70	KF246087	14-JUN-2013	*Klebsiella pneumoniae*	Unknown	*bla*$_{\text{CTX-M-15}}$	Chennai, Tamil Nadu
71	KF246088	14-JUN-2013	*Klebsiella pneumoniae*	Unknown	*bla*$_{\text{CTX-M-15}}$	Chennai, Tamil Nadu
72	KC699838	28-FEB-2013	*Klebsiella pneumoniae*	Urine	*bla*$_{\text{CTX-M-15}}$	Kolkata, West Bengal
73	KC528758	18-JAN-2013	*Klebsiella pneumoniae*	Contact lens	*bla*$_{\text{CTX-M-15}}$	Chennai, Tamil Nadu
74	KC528759	18-JAN-2013	*Klebsiella pneumoniae*	Contact lens	*bla*$_{\text{CTX-M-15}}$	Chennai, Tamil Nadu
75	KC528760	18-JAN-2013	*Klebsiella pneumoniae*	Conjunctival swab	*bla*$_{\text{CTX-M-15}}$	Chennai, Tamil Nadu
76	KC528761	18-JAN-2013	*Klebsiella pneumoniae*	Corneal rim	*bla*$_{\text{CTX-M-15}}$	Chennai, Tamil Nadu
77	KC528762	18-JAN-2013	*Klebsiella pneumoniae*	Contact lens	*bla*$_{\text{CTX-M-15}}$	Chennai, Tamil Nadu
78	KC528770	18-JAN-2013	*Klebsiella pneumoniae*	Eviscerated material	*bla*$_{\text{CTX-M-15}}$	Chennai, Tamil Nadu
79	KC528771	18-JAN-2013	*Klebsiella pneumoniae*	Orbital biopsy	*bla*$_{\text{CTX-M-15}}$	Chennai, Tamil Nadu
80	KC143066	06-NOV-2012	*Klebsiella pneumoniae*	Urine	*bla*$_{\text{CTX-M-15}}$	Chennai, Tamil Nadu
81	KC153031	08-NOV-2012	*Klebsiella pneumoniae*	Conjunctival swab	*bla*$_{\text{CTX-M-15}}$	Tirunelveli, Tamil Nadu
82	JQ235795	05-DEC-2011	*Klebsiella pneumoniae*	Eviscerated material	*bla*$_{\text{CTX-M-15}}$	Chennai, Tamil Nadu
83	JQ235797	05-DEC-2011	*Klebsiella pneumoniae*	Orbital biopsy	*bla*$_{\text{CTX-M-15}}$	Chennai, Tamil Nadu
84	JQ235800	05-DEC-2011	*Klebsiella pneumoniae*	Vitreous humour	*bla*$_{\text{CTX-M-15}}$	Chennai, Tamil Nadu
85	JQ235803	05-DEC-2011	*Klebsiella pneumoniae*	Donor corneal rim	*bla*$_{\text{CTX-M-15}}$	Chennai, Tamil Nadu
86	JQ235805	05-DEC-2011	*Klebsiella pneumoniae*	Conjunctival swab	*bla*$_{\text{CTX-M-15}}$	Chennai, Tamil Nadu
87	JN019838	24-MAY-2011	*Klebsiella pneumoniae*	Corneal rim	*bla*$_{\text{CTX-M-15}}$	Chennai, Tamil Nadu
88	JN019840	24-MAY-2011	*Klebsiella pneumoniae*	Corneal rim	*bla*$_{\text{CTX-M-15}}$	Chennai, Tamil Nadu
89	JN019841	24-MAY-2011	*Klebsiella pneumoniae*	Corneal rim	*bla*$_{\text{CTX-M-15}}$	Chennai, Tamil Nadu
90	JN019843	24-MAY-2011	*Klebsiella pneumoniae*	Corneal rim	*bla*$_{\text{CTX-M-15}}$	Chennai, Tamil Nadu
91	JN019844	24-MAY-2011	*Klebsiella pneumoniae*	Corneal rim	*bla*$_{\text{CTX-M-15}}$	Chennai, Tamil Nadu
92	JN019846	24-MAY-2011	*Klebsiella pneumoniae*	Viterous chamber tap	*bla*$_{\text{CTX-M-15}}$	Chennai, Tamil Nadu
93	JN019847	24-MAY-2011	*Klebsiella pneumoniae*	Corneal rim	*bla*$_{\text{CTX-M-15}}$	Chennai, Tamil Nadu
94	JN019849	24-MAY-2011	*Klebsiella pneumoniae*	Conjunctival swab	*bla*$_{\text{CTX-M-15}}$	Chennai, Tamil Nadu

95	JN019850	24-MAY-2011	*Klebsiella pneumoniae*	Conjunctival swab	$bla_{CTX-M-15}$	Chennai, Tamil Nadu
96	JN019851	24-MAY-2011	*Klebsiella pneumoniae*	Corneal scraping	$bla_{CTX-M-15}$	Chennai, Tamil Nadu
97	JN019852	24-MAY-2011	*Klebsiella pneumoniae*	Aqueous chamber tap	$bla_{CTX-M-15}$	Chennai, Tamil Nadu
98	JN019855	24-MAY-2011	*Klebsiella pneumoniae*	Corneal rim	$bla_{CTX-M-15}$	Chennai, Tamil Nadu
99[1]	AB545871	04-FEB-2010	*Klebsiella pneumoniae*	Neonatal stool	$bla_{CTX-M-14}$	Kolkata, West Bengal
100	EU979558	01-JUL-2008	*Klebsiella pneumoniae*	Endotracheal secretion	$bla_{CTX-M-15}$	Chennai, Tamil Nadu
101	EU531511	23-FEB-2008	*Klebsiella pneumoniae*	Urine	$bla_{CTX-M-15}$	Chennai, Tamil Nadu
102	EU531512	23-FEB-2008	*Klebsiella pneumoniae*	Endotracheal secretion	$bla_{CTX-M-15}$	Chennai, Tamil Nadu
103	KC528767	18-JAN-2013	*Enterobacter aerogenes*	Aqueous humor	$bla_{CTX-M-15}$	Chennai, Tamil Nadu
104	KC528768	18-JAN-2013	*Enterobacter aerogenes*	Vitreous humor	$bla_{CTX-M-15}$	Chennai, Tamil Nadu
105	KC528769	18-JAN-2013	*Enterobacter aerogenes*	Corneal rim	$bla_{CTX-M-15}$	Chennai, Tamil Nadu
106	KC200079	18-NOV-2012	*Enterobacter aerogenes*	Corneal rim	$bla_{CTX-M-15}$	Tirunelveli, Tamil Nadu
107	JQ235794	05-DEC-2011	*Enterobacter aerogenes*	Corneal rim	$bla_{CTX-M-15}$	Chennai, Tamil Nadu
108	JQ235799	05-DEC-2011	*Enterobacter aerogenes*	Contact lens	$bla_{CTX-M-15}$	Chennai, Tamil Nadu
109	JN019835	24-MAY-2011	*Enterobacter aerogenes*	Conjunctival swab	$bla_{CTX-M-15}$	Chennai, Tamil Nadu
110	EU979557	01-JUL-2008	*Enterobacter* sp. O050109	Endotracheal secretion	$bla_{CTX-M-15}$	Chennai, Tamil Nadu
111	EU531513	23-FEB-2008	*Enterobacter* sp. O050109	Endotracheal secretion	$bla_{CTX-M-15}$	Chennai, Tamil Nadu
112	KC528772	18-JAN-2013	*Klebsiella oxytoca*	Orbital biopsy	$bla_{CTX-M-15}$	Chennai, Tamil Nadu
113	JQ235798	05-DEC-2011	*Klebsiella oxytoca*	Orbital biopsy	$bla_{CTX-M-15}$	Chennai, Tamil Nadu
114	JQ235801	05-DEC-2011	*Klebsiella oxytoca*	Corneal rim	$bla_{CTX-M-15}$	Chennai, Tamil Nadu
115	JQ235804	05-DEC-2011	*Klebsiella oxytoca*	Conjunctival swab	$bla_{CTX-M-15}$	Chennai, Tamil Nadu
116	JN019839	24-MAY-2011	*Klebsiella oxytoca*	Corneal rim	$bla_{CTX-M-15}$	Chennai, Tamil Nadu
117	JN019842	24-MAY-2011	*Klebsiella oxytoca*	Corneal rim	$bla_{CTX-M-15}$	Chennai, Tamil Nadu
118	KC528765	18-JAN-2013	*Proteus mirabilis*	Corneal scraping	$bla_{CTX-M-15}$	Chennai, Tamil Nadu
119	JQ235796	05-DEC-2011	*Proteus mirabilis*	Corneal scraping	$bla_{CTX-M-15}$	Chennai, Tamil Nadu
120	JQ235806	05-DEC-2011	*Proteus mirabilis*	Corneal scraping	$bla_{CTX-M-15}$	Chennai, Tamil Nadu
121	JN019836	24-MAY-2011	*Proteus mirabilis*	Conjunctival swab	$bla_{CTX-M-15}$	Chennai, Tamil Nadu
122	JN019854	24-MAY-2011	*Proteus mirabilis*	Corneal rim	$bla_{CTX-M-15}$	Chennai, Tamil Nadu
123	JQ235802	05-DEC-2011	*Citrobacter koseri*	Vitreous humour	$bla_{CTX-M-15}$	Chennai, Tamil Nadu
124	JN019837	24-MAY-2011	*Citrobacter koseri*	Corneal rim	$bla_{CTX-M-15}$	Chennai, Tamil Nadu
125	JN019848	24-MAY-2011	*Citrobacter koseri*	Corneal rim	$bla_{CTX-M-15}$	Chennai, Tamil Nadu
126	JN019856	24-MAY-2011	*Citrobacter koseri*	Conjunctiva	$bla_{CTX-M-15}$	Chennai, Tamil Nadu

127	JN019853	24-MAY-2011	*Citrobacter freundii*	Eviscerated material	$bla_{CTX-M-15}$	Chennai, Tamil Nadu
128	JN019857	24-MAY-2011	*Citrobacter freundii*	Conjunctiva	$bla_{CTX-M-15}$	Chennai, Tamil Nadu
129	HM117627	11-APR-2010	*Salmonella enterica*	Blood sample	$bla_{CTX-M-15}$	Chennai, Tamil Nadu
130	KC200078	18-NOV-2012	*Pantoea agglomerans*	Corneal rim	$bla_{CTX-M-15}$	Tirunelveli, Tamil Nadu
131	KC528766	18-JAN-2013	*Serratia marcescens*	Corneal scraping	$bla_{CTX-M-15}$	Chennai, Tamil Nadu
132	KR824153	15-MAY-2015	*Pseudomonas aeruginosa*	Corneal button	$bla_{CTX-M-15}$	Chennai, Tamil Nadu
133	KR824154	15-MAY-2015	*Pseudomonas aeruginosa*	Eviscerated material	$bla_{CTX-M-15}$	Chennai, Tamil Nadu
134	AAL02127	09-JUL-2001	Unknown	Unknown	$bla_{CTX-M-15}$	New Delhi, Delhi

[1]Sequences that were not $bla_{CTX-M-15}$
